# The Impact of Oxidative Stress in Male Infertility

**DOI:** 10.3389/fmolb.2021.799294

**Published:** 2022-01-05

**Authors:** Amanda Mannucci, Flavia Rita Argento, Eleonora Fini, Maria Elisabetta Coccia, Niccolò Taddei, Matteo Becatti, Claudia Fiorillo

**Affiliations:** ^1^ Department of Experimental and Clinical Biomedical Sciences “Mario Serio”, University of Florence, Florence, Italy; ^2^ Assisted Reproductive Technology Centre, Careggi Hospital, University of Florence, Florence, Italy

**Keywords:** oxidative stress, ROS, reactive oxygen species, male infertility, spermatozoa, semen parameters

## Abstract

At present infertility is affecting about 15% of couples and male factor is responsible for almost 50% of infertility cases. Oxidative stress, due to enhanced Reactive Oxygen Species (ROS) production and/or decreased antioxidants, has been repeatedly suggested as a new emerging causative factor of this condition. However, the central roles exerted by ROS in sperm physiology cannot be neglected. On these bases, the present review is focused on illustrating both the role of ROS in male infertility and their main sources of production. Oxidative stress assessment, the clinical use of redox biomarkers and the treatment of oxidative stress-related male infertility are also discussed.

## Introduction

Infertility is a multifactorial disease affecting 15% of couples and defined as the inability to achieve spontaneous pregnancy after 12 months or more of regular unprotected sexual intercourse (Sharlip et al., 2002; Aitken, 2020). Male factor is responsible for almost 50% of infertility cases, contributing equally as female factor (Athayde et al., 2007; Wagner et al., 2018). Male infertility diagnosis is commonly based on standard semen parameters analysis (Nallella et al., 2006), according to the WHO guidelines, nevertheless, a large proportion of infertile males does not receive a clear diagnosis, considering them as idiopathic or unexplained cases (Sharlip et al., 2002).

Many studies suggested oxidative stress, a condition characterized by an imbalance between reactive oxygen species (ROS) production and antioxidant defence systems, as a new emerging factor in unexplained male infertility (Saleh and Agarwal, 2002; Makker et al., 2009; Agarwal et al., 2018; Cito et al., 2020).

At physiological levels, ROS are associated with the development of sperm fertilization properties, promoting chromatin compaction in maturing spermatozoa, motility, chemotaxis, sperm capacitation, hyperactivation, acrosome reaction and oocyte interaction (Kothari et al., 2010; Du Plessis et al., 2015). An excessive ROS production represents an important cause of sperm injury. Indeed, due to the large amount of membrane unsaturated fatty acids and the lack of cytoplasmic antioxidant enzymes, spermatozoa are highly susceptible to oxidation (Agarwal et al., 2017), with consequent detrimental effects on sperm quality/functioning (Aitken and Baker, 2006; Venkatesh et al., 2011; Barati et al., 2020).

Here, we discuss about the different roles of ROS on spermatozoa pathophysiology, paying particular attention to ROS effects on semen parameters. Finally, we focus on the available techniques to assess redox status in biological fluids and the clinical use of redox biomarkers for diagnosis and management of male infertility.

### Oxidative Stress

Oxygen has a central role in life, displaying both beneficial and harmful effects on biological systems. The main oxygen involvement is in adenosine-5-triphosphate (ATP) generation via mitochondrial oxidative phosphorylation (Burton and Jauniaux, 2011; Lushchak, 2014), a reaction also implicated in ROS and RNS production (Pham-Huy et al., 2008).

At moderate levels, ROS/RNS play an important role in regulating several intracellular signaling pathways, immune and mitogen responses and in maintaining cellular homeostasis (Kruk et al., 2019). On the contrary, higher ROS levels can be responsible for oxidative damages on proteins, lipids and nucleic acids (DNA, RNA), with harmful cellular effects. However, a complex system of antioxidant molecules has been evolved to maintain a redox balance and avoid biological system injury (Burton and Jauniaux, 2011; Kruk et al., 2019).

Several conditions (as environmental factors, excessive physical exercise, deficiencies in antioxidants, immune system dysfunctions, chronic disorders) may alter oxidant/antioxidant balance, leading to oxidative stress (Halliwell, 2007).

Oxidative stress mediates tissue injury and cell death, displaying a pathological role in several disorders including inflammation and aging, cardiovascular and neurodegenerative diseases, autoimmune disorders, cancer and reproductive system alterations (Burton and Jauniaux, 2011; Birben et al., 2012; Kruk et al., 2019).

### The Physiological Role of ROS in Spermatozoa

Physiologically, ROS are considered regulators of several intracellular pathways, modulating the activation of different transcription factors (Burton and Jauniaux, 2011). ROS stimulate cyclic adenosine monophosphate (cAMP) in sperms, promoting tyrosine phosphorylation by tyrosine phosphatase inhibition (Wagner et al., 2018). This molecular mechanism results in the activation of several transcription factors involved in intracellular signaling cascades for sperm physiology. Indeed, several studies showed that higher ROS levels stimulate sperm capacitation and hyperactivation, acrosome reaction, motility and chemotaxis and chromatin compaction in maturing spermatozoa (Du Plessis et al., 2015; Wagner et al., 2018). Furthermore, ROS can improve sperm capacity of binding to the zona pellucida, inducing sperm-oocyte fusion (Wagner et al., 2018). By the way, antioxidant molecules may alter spermatozoa maturation, interfering with physiological sperm function. Particularly, it was showed that catalase or superoxide dismutase (SOD) inhibit sperm capacitation or acrosome reaction, supporting the evidence of the central involvement of ROS in spermatozoa functioning (Wagner et al., 2018).

### The Pathological Role of ROS in Spermatozoa

Besides to the physiological role of ROS, excessive ROS generation and oxidative stress seem to be associated with harmful effects on spermatozoa, resulting in morphological and dynamic cellular properties alterations and finally in lower fertilization ability.

During recent years, a growing literature has shown that an altered redox balance in seminal fluid may display deleterious effects on sperm homeostasis, leading to male infertility (Agarwal et al., 2008; Makker et al., 2009; Agarwal et al., 2014b; Sabeti et al., 2016; Agarwal et al., 2017; Agarwal et al., 2018; Majzoub and Agarwal, 2018).

Blood and plasma redox status alterations have been reported in infertile men, as recently described in a study (Cito et al., 2020) showing higher blood leukocytes ROS production, increased plasma lipid peroxidation (LPO) and reduced plasma total antioxidant capacity (TAC) in oligoasthenozoospermic men compared to healthy subjects (Cito et al., 2020). In line with this, several findings also suggest that ROS-mediated sperm oxidation may induce cellular dysfunctions, affecting spermatozoa concentration, total number and motility (Agarwal et al., 2008; Agarwal et al., 2014b).

Spermatozoa are particularly susceptible to ROS-induced oxidation due to the presence, in their plasma membrane, of elevated levels of polyunsaturated fatty acids as docosahexaenoic acid containing six double bonds per molecule (Aitken et al., 2014). Indeed, ROS mediate the hydrogen abstraction from the hydrocarbon side-chain of a fatty acid, yielding to a carbon-centered lipid radical (L·) whose interaction with oxygen produces a lipid peroxyl radical (LOO·), able to react with an adjacent fatty acid propagating the process. Following internal molecular rearrangements conjugated dienes and hydroperoxides are generated (Phaniendra et al., 2015; Yoshida et al., 2015).

LPO products can also react with proteins, DNA and phospholipids, generating end-products involved in cellular dysfunction. Particularly, the interaction of LPO products with amino residues can result in protein oxidation, affecting protein structural and functional features (Niki, 2014). In this context, it was observed that LPO products as 4-hydroxy-2-nonenal (4HNE) are able to propagate ROS generation via interaction with proteins of the sperm mitochondrial electron transport chain (Aitken et al., 2014).

Lipid peroxidation is strictly associated with fluidity and permeability membrane alterations, inhibition of membrane-bound enzymes and receptors and activation of apoptotic cascade, supporting oxidative stress involvement in motility and morphology sperms abnormalities (Nowicka-Bauer and Nixon, 2020). Among LPO products, 4HNE seems to be highly responsible for cytotoxic effects on cellular sperm membrane, inducing loss of membrane integrity, motility alterations and compromising sperm-oocyte interactions (Baker et al., 2015; Walters et al., 2018; Nowicka-Bauer and Nixon, 2020) It was observed that 4HNE-mediated effects depends on several factors: cellular differentiation status, amount of substrates for 4HNE attack and antioxidant defense systems (Walters et al., 2018).

ROS can also affect sperm functioning by post-translational oxidative protein modifications (Salvolini et al., 2012; Morielli and O'Flaherty, 2015). The important association between protein oxidation markers, as three nitro-tyrosines (3NT), and sperm motility and morphology in oligoasthenoteratospermia has been reported (Kalezic et al., 2018). In particular, signs of sperm protein S-glutathionylation and tyrosine nitration were found in infertile men (Salvolini et al., 2012; Morielli and O'Flaherty, 2015). Accordingly, higher peroxynitrite levels in human asthenozoospermic sperm samples, emphasizing their negative impact on sperm motility through the formation of three nitro-tyrosines were reported (Vignini et al., 2006).

Several investigations observed that not all sperm proteins are equally susceptible to ROS or to lipid aldehydes (Nowicka-Bauer and Nixon, 2020). The principal 4HNE target proteins are represented by metabolic enzymes, involved in bioenergetic pathways needed for sperm motility (Moscatelli et al., 2019). Several proteomics studies have been performed on infertile men spermatozoa in this context. A downregulation of proteins involved in bioenergetic pathways in altered spermatozoa of asthenozoospermic men was revealed (Amaral et al., 2014; Moscatelli et al., 2019). Particularly, some authors observed alterations in proteins associated with metabolic pathways as glycolysis, pyruvate metabolism, TCA or beta-oxidation in asthenozoospermic men, supporting that oxidative stress compromises sperm functionality by altering bioenergetic pathways (Elkina et al., 2011; Guo et al., 2019).

It is traditionally accepted that nucleic acids represent another crucial target of oxidative stress. Both nuclear and mitochondrial DNA are vulnerable to hydroxyl radical (OH.) attack, leading to the formation of several biomarkers of oxidative stress. OH. can react with guanine to produce 8-hydroxy-2′-deoxyguanosine (8-OH-G), an important marker of DNA oxidative damage, detectable in several biological samples (Burton and Jauniaux, 2011).

The lack of adequate antioxidant systems makes spermatozoa highly susceptible to DNA oxidation (Agarwal et al., 2003; Aitken et al., 2014). Sperm DNA oxidation is also due to the lack of complete DNA repair strategies in spermatozoa. Indeed, if the 8-oxoguanine glycosylase (OGG1) is able to remove the 8OHdG residue from DNA producing an abasic site, sperms do not possess any base excision repair system for the insertion of a new base (Aitken et al., 2014).

Several studies indicated that ROS generation is associated with DNA fragmentation and poor chromatin packaging, promoting apoptosis with relevant consequences on sperm count (Aitken et al., 2014; Iommiello et al., 2015). Patients with asthenozoospermia show enhanced mtDNA copy number and reduced mtDNA integrity that are associated with higher ROS generation (Bonanno et al., 2016). Accordingly, other reports underlined the significant association between NO and 8-OHdG levels and semen parameters abnormalities (Gholinezhad et al., 2020), supporting redox status assessment for helping male infertility diagnosis and monitoring.

## Main Sources of ROS

It is largely accepted that several exogenous factors may contribute to inflammation and redox status alterations, promoting male infertility. Environmental pollution, lifestyle factors as smoke, alcohol, obesity, varicocele, bacterial/viral infections, microorganism mutations or sexual transmitted disorders are actively involved (Iommiello et al., 2015; Agarwal et al., 2018).

However, seminal fluid oxidative stress is mostly due to leukocytes -that produce 1,000 more times ROS than normal spermatozoa- and to immature spermatozoa (Agarwal et al., 2003; Iommiello et al., 2015; Agarwal et al., 2018).

**Leukocytospermia.** According to WHO guidelines, leukocytospermia, defined as peroxidase-positive leukocytes concentration >1 × 106 per mL of semen, has been found in about 10–20% of infertile men (Saleh et al., 2002; Agarwal et al., 2018). Granulocytes and macrophages are the main cellular types found in the ejaculate and are responsible for ROS generation which is largely associated to glucose-6-phosphate dehydrogenase (G6PDH) activity, producing high amount of NADPH that, in turn, strongly stimulates NADPH oxidase, one of the major ROS sources (Agarwal et al., 2003; Agarwal et al., 2018). New emerging observations revealed that seminal WBC could improve sperm ability to generate ROS in a direct manner or by soluble products released in sperm microenvironment (Saleh et al., 2002). However, the clinical significance of leukocytospermia and its role in sperm quality is still under debate.

Higher seminal WBC levels were observed in infertile men compared to healthy controls and leukocytospermia was significantly correlated with alterations in sperm number, motility and morphology (Wolff, 1995). Moreover, *in vitro* experiments showed that WBC damaged sperm function and hamster ovum penetration, representing important prognostic factors for Assisted Reproductive Technologies (ART) success rate (Wolff, 1995). In line with this evidence, further investigations supported WBC as a trigger factor for spermatozoa ROS generation, leading to reduced sperm quality and sperm DNA damage (Saleh et al., 2002; Agarwal et al., 2014a). Leukocytospermia was associated with alterations in sperm concentration, motility and morphology in leukocytospermic patients respect to nonleukospermic patients or healthy subjects. *In vitro* experiments also underlined that ROS levels remained increased in pure sperms suspensions of leukocytospermic patients also after WBC removal or phorbol 12-myristate 13-acetate (PMA)-induced ROS stimulation. Similar results were obtained after sperm incubation with WBC (Saleh et al., 2002).

Moreover, semen WBC, even at low concentrations, resulted positively correlated with oxidative stress, suggesting that semen WBC removal could be useful to reduce oxidative stress in samples used for ART (Sharma et al., 2001; Agarwal et al., 2014a).

**Immature spermatozoa.** When spermatogenesis is defective, alterations in cytoplasmic extrusion mechanisms are observed and spermatozoa are released with an excess of residual cytoplasm (cytoplasmic droplets) (Agarwal et al., 2003). Immature spermatozoa are associated with higher ROS generation, via G6PDH and higher creatine phosphokinase (CK) levels (Cayli et al., 2004). The clinical significance of CK in sperm maturity and quality is controversial (Hallak et al., 2001; Cayli et al., 2004; Muratori et al., 2015). Some reports described higher CK levels in oligozoospermic men than in healthy subjects and a significant association between CK levels and semen parameters (concentration, motility and morphology), suggesting this marker as a good predictor of sperm quality in the follow-up of patients treated for male infertility (Hallak et al., 2001). Other authors found no difference in CK amount between cells with or without DNA fragmentation, showing no involvement of immature spermatozoa in DNA damage (Muratori et al., 2015). In this context, it was observed that spermatozoa at different stages of maturation are characterized by variations in ROS levels, membrane lipid content, chromatin compaction, morphology and motility. Immature spermatozoa showed higher ROS generation and DNA damage and could be considered an important cause of male infertility, inducing oxidation in mature sperm cells during sperm migration from the seminiferous tubules to the epididymis (Ollero et al., 2001).

**Mithocondria.** Another potential ROS source in spermatozoa is represented by mitochondria. Indeed, factors as electromagnetic radiation, polyunsaturated fatty acids or apoptotic factors may alter the electron transport chain on mitochondrial membrane, resulting in excessive ROS generation. Several reports indicate sperm mitochondrial dysfunction and oxidative stress as potential factors involved in asthenozoospermia (Nowicka-Bauer et al., 2018). Particularly, interferences in the mitochondrial electron flow at complexes I and III may trigger ROS generation and cause sperm tail oxidation, leading to DNA damage and motility aberrations (Koppers et al., 2008). Sperm mitochondrial dysfunctions enhance ROS production and are associated with sperm quality impairment and loss of fertilization potential. Particularly, a significant correlation between sperm mitochondrial functioning and sperm motility was reported (Cassina et al., 2015).

## Oxidative Stress Effects on Semen Parameters

**FIGURE 1:**
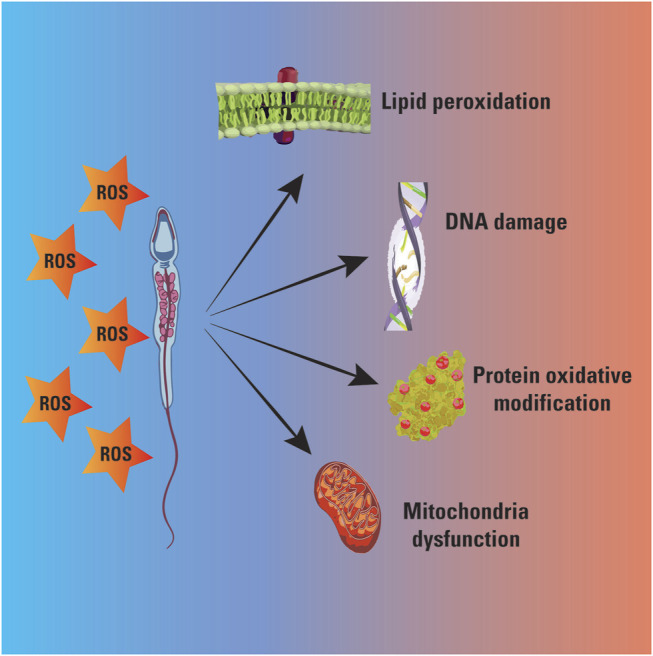
Oxidative stress negatively affects sperm cells causing mitochondrial injury and alterations in lipids, nucleic acids and proteins.

During these years, the potential correlation between spermatozoa ROS production and semen parameters has been largely investigated (Athayde et al., 2007; Hosseinzadeh Colagar et al., 2013; Agarwal et al., 2014a; Bonanno et al., 2016; Aitken, 2017; Dobrakowski et al., 2017; Dorostghoal et al., 2017). The detrimental effects of ROS on sperm motility and morphology has been repeatedly reported. *In vitro* experiments demonstrated that lipid aldheydes addiction to spermatozoa promoted loss motility in human sperm cells (Agarwal et al., 2014a) (Figure 1).

Accordingly, seminal fluid LPO and TAC levels were significantly correlated with sperm motility, morphology and sperm count in astheno- and oligoastheno-teratospermic men (Khosrowbeygi and Zarghami, 2007; Hosseinzadeh Colagar et al., 2013).

The key role of oxidative stress in spermatozoa alterations is also supported by evidence of beneficial effects of therapeutic supplementation with antioxidants on semen quality in infertile men (Gambera et al., 2019). In particular, therapeutic Coenzyme Q10 treatment improved semen parameters (sperm concentration and motility), redox status and sperm DNA fragmentation in idiopathic male infertility (Alahmar et al., 2021). Interestingly, an improvement in sperm concentration and motility after vitamin D supplementation in vitamin D deficient infertile male with oligoasthenozoospermia was observed (Wadhwa et al., 2020). The positive effects of an antioxidant therapy (Gambera et al., 2019) on semen quality has been suggested as a useful tool to improve successful conception rate in patients with oligoasthenozoospermia undergoing intracytoplasmic sperm injection (ICSI).

On the contrary, other authors reported no correlation between ROS levels and sperm motility, underling that it is still unclear if reduced sperm functional performances are due to lower sperm number or to a direct ROS effect (Whittington et al., 1999).

In this context, the usefulness of a new blood diagnostic tool to evaluate sperm morphological and/or functional abnormalities, supporting male infertility diagnosis and management, is increasingly evident.

In this regards, blood SOD and GSH levels were found to positively correlate with sperm count and motility, while enhanced MDA levels were associated with altered sperm morphology (Shamsi et al., 2010). In line with this, signs of oxidative stress in seminal fluid and reduced plasma TAC in infertile men were described. Particularly, plasma TAC significantly and positively correlated both with seminal fluid TAC and with semen parameters (Benedetti et al., 2012), indicating that plasma redox status reflects the redox status of seminal fluid microenvironment and sperm quality.

In agreement, it has been shown that higher MDA and Nitric Oxide (NO) levels in plasma and seminal fluid of infertile men correlated with semen parameters, supporting that blood redox status is associated with semen parameters (Taken et al., 2016).

However, reports about the existing association between blood and seminal fluid oxidative stress are still limited and controversial, potentially due to different strategies and applied methodologies. Indeed, no correlation was found between blood and seminal fluid oxidative status, suggesting the independence of seminal fluid redox homeostasis from systemic microenvironment and external factors (Guz et al., 2013).

## Oxidative Stress Assessment and Clinical Use of Redox Biomarkers in Male Infertility

The analysis of semen parameters according to the WHO guidelines represents, currently, the gold standard for male infertility diagnosis. However, several studies showed that ROS-induced sperm oxidation can result in sperm quality alterations, leading to a decrease in sperm fertilizing potential (Agarwal and Majzoub, 2017; Dutta et al., 2019; Martins and Agarwal, 2019). Based on this evidence, new tests aimed to evaluate male fertility by monitoring oxidative stress status are needed.

Assays for oxidative stress detection may suggest new biochemical approaches to improve male infertility diagnosis and management, using simple, fast and less expensive techniques (Agarwal and Majzoub, 2017; Agarwal et al., 2019).

Oxidative stress can be evaluated in different biological samples (plasma, serum, urine, follicular/peritoneal/seminal fluid), obtaining an accurate picture of redox status and eventually planning a therapeutic supplementation with antioxidants where it’s needed.

Different oxidative stress assays exist, focusing on ROS generation, lipid peroxidation products and total antioxidant capacity. ROS measurement in semen include different methods as chemiluminescence, nitro blue tetrazolium (NBT) test, cytochrome c reduction test and electron spin resonance (Dutta et al., 2019; Martins and Agarwal, 2019).

However, several reports underlined the central use of cytometry to assess intracellular ROS production in blood cells as erythrocytes and leucocytes, in spermatozoa as well as in other cellular categories by incubating cells with the fluorescent probe H2DCF-DA (2.5 µM) (Invitrogen, Carlsbad, CA, United States) (Becatti et al., 2016a; Becatti et al., 2017a; Becatti et al., 2018; Cito et al., 2020). Due to its susceptibility to ROS-induced oxidation by hydrogen peroxide, peroxynitrite, hydroxyl radicals and also by superoxide anions, H2DCF-DA is now considered among the principal methods for measuring intracellular ROS levels, sensing redox status variations and cellular oxidative stress (Eruslanov and Kusmartsev, 2010).

Cytofluorimetric analysis can be also employed for the assessment of membrane lipid oxidation, using the fluorescent probe BODIPY 581/591 C11. This approach was proposed to investigate redox status alterations both in erythrocytes of RVO (Becatti et al., 2016b) and SSNHL (Becatti et al., 2017b) patients.

Moreover, fluorescent anisotropy of cellular membranes, a new method to evaluate membrane fluidity, could be a future innovation for further investigations about sperm motility defects (Becatti et al., 2016b; Becatti et al., 2017b).

Oxidative stress assessment is also performed by evaluating LPO and TAC levels in biological fluids (Martins and Agarwal, 2019). LPO levels can be detected by measuring lipid oxidation end products as MDA, 4HNE, isoprostanes with spectrofluorimetric or immunochemical assays (Agarwal and Majzoub, 2017). Thiobarbituric Acid (TBA) Assay or the ALDETECT Assay are the mostly used tests for LPO assessment. Highly sensitive high pressure liquid chromatography (HPLC) is promoted for low MDA concentrations (Grotto et al., 2007), whereas commercial immunoassays or mass spectrometry represent an alternative method to evaluate lipid peroxidation end products as isoprostanes (Morrow, 2005).

Parallelly, TAC level can be measured using enhanced-chemiluminescence or colorimetric techniques (Martins and Agarwal, 2019). Among chemiluminescent methodologies, Oxygen Radical Absorbance Capacity (ORAC) Assay is based on the intensity fluorescence decay of a fluorescent probe, fluorescein, consequent to its oxidation by free radical species (particularly peroxyl radical), generated after the thermal decomposition of 2,2′-azobis (2-amidinopropane) dihydrochloride (AAPH) azo-compound. Colorimetric methods evaluate the antioxidant capacity of samples to inhibit the oxidation of 2,2′-azino-bis(3-ethylbenzothiazoline- 6-sulphonic acid) (ABTS) to ABTS + by metmyoglobin (Martins and Agarwal, 2019).

Based on redox biomarkers alterations in infertile men, several studies emphasized the elaboration of a specific global parameter/index able to discriminate fertile from infertile men better than ROS or TAC alone (Roychoudhury et al., 2016). Particularly, ROS-TAC score, derived from both ROS levels and antioxidant capacity in a given set of patients, was proposed as a new tool to investigate redox status in male infertility. Infertile men with male factor or idiopathic diagnoses had significantly different ROS-TAC scores than controls (Sharma et al., 1999; Vatannejad et al., 2017). Particularly, the potential use of ROS-TAC score for predicting the oxidative damage of semen samples in asthenozoospermic men was proposed (Vatannejad et al., 2017).

New emerging data have also shown oxidation reduction potential (ORP) measurement as a new fast, easy and reproducible method to assess oxidative stress in seminal fluid (Agarwal and Majzoub, 2017; Martins and Agarwal, 2019).

ORP indicates the ratio between oxidant and antioxidant molecules, evaluating the potential for electrons to move from a chemical specie to another. ORP is assessed by MiOSYS test, that measures electron transfer from antioxidants to oxidants in presence of a low voltage reducing current. The obtained data represent oxidant and antioxidant activity in a sample: particularly, high ORP levels indicate enhanced oxidant activity and therefore a condition of oxidative stress (Agarwal and Majzoub, 2017).

Some evidence reports a good association between ORP level and semen parameters (Agarwal et al., 2017; Majzoub et al., 2018; Homa et al., 2019) being found higher ORP levels in infertile men than in healthy controls (Agarwal et al., 2017). Moreover, a negative correlation between ORP value and semen parameters (sperm concentration and total count, motility and morphology) was observed suggesting ORP as a further predictor for male infertility diagnosis and management (Agarwal et al., 2017). In line with this, further investigations confirmed the significant association of ORP both with semen parameters and DNA fragmentation in infertile men (Majzoub et al., 2018; Homa et al., 2019). Importantly, it was also shown that ORP is a more accurate tool for investigating redox status in male infertility than chemiluminescent ROS assessment (Homa et al., 2019).

## Treatment of Oxidative Stress Related Male Infertility

Currently, defined guidelines for treatment of oxidative stress-related male infertility are still lacking, in partly due to the unknown etiology of this condition (Agarwal et al., 2019). However, during these years several clinical trials have been developed to investigate the effects of antioxidant supplementation (as l-carnitine, selenium, Coenzyme Q10, ubiquinol, vitamin C and E) on seminal fluid oxidative stress and semen parameters (Majzoub and Agarwal, 2018; Dutta et al., 2019). Many of them reported promising effects of antioxidants on sperm concentration, motility, morphology and DNA fragmentation (Gambera et al., 2019; Alahmar et al., 2021). Twenty clinical trials focused on antioxidant therapy effects on seminal oxidative stress were analyzed. Nineteen of them revealed an improvement in sperm redox status and semen parameters and a good correlation with pregnancy outcome (Gharagozloo and Aitken, 2011).

However, the role of antioxidant therapy in male infertility is still controversial. In a randomized clinical trial, it was showed that 3 months of antioxidant treatment did not improve semen parameters and DNA fragmentation in infertile men and no beneficial effect on pregnancy or live birth rates was observed (Steiner et al., 2020).

These observations indicate that evidence to support the use of antioxidants in male infertility are still uncertain. However, traditional semen analysis together with oxidative stress assessment display a great potential to perform accurate evaluation of infertile patients (Agarwal et al., 2019).

## Conclusion

Oxidative stress is centrally involved in sperm dysfunctions and represent a new pathological mechanism of male infertility (Agarwal et al., 2008; Hosseinzadeh Colagar et al., 2013; Agarwal et al., 2018). Based on previously reported investigations and results, new methods and diagnostic approaches for male reproductive disorders are needed. Together with seminal fluid oxidative stress assessment, blood redox status monitoring and leukocytes ROS levels, could represent a new potential and less invasive practice for clinicians to evaluate sperm cells quality and fertilization ability. The considered redox parameters may therefore be useful to develop new therapeutic strategies based on antioxidant supplementation in order to reduce systemic oxidative stress in infertile men, improving male infertility diagnosis and management and ART success rate.

## References

[B1] Agarwal A. Majzoub A. (2017). Laboratory Tests for Oxidative Stress. Indian J. Urol. 33 (3), 199–206. 10.4103/iju.IJU_9_17 28717269 PMC5508430

[B2] Agarwal A. Saleh R. A. Bedaiwy M. A. (2003). Role of Reactive Oxygen Species in the Pathophysiology of Human Reproduction. Fertil. Sterility 79 (4), 829–843. 10.1016/s0015-0282(02)04948-8 12749418

[B3] Agarwal A. Makker K. Sharma R. (2008). Review Article: Clinical Relevance of Oxidative Stress in Male Factor Infertility: An Update. Am. J. Reprod. Immunol. 59 (1), 2–11. 10.1111/j.1600-0897.2007.00559.x 18154591

[B4] Agarwal A. Mulgund A. Alshahrani S. Assidi M. Abuzenadah A. M. Sharma R. (2014). Reactive Oxygen Species and Sperm DNA Damage in Infertile Men Presenting with Low Level Leukocytospermia. Reprod. Biol. Endocrinol. 12, 126. 10.1186/1477-7827-12-126 25527074 PMC4292986

[B5] Agarwal A. Virk G. Ong C. du Plessis S. S. (2014). Effect of Oxidative Stress on Male Reproduction. World J. Mens Health 32 (1), 1–17. 10.5534/wjmh.2014.32.1.1 24872947 PMC4026229

[B6] Agarwal A. Roychoudhury S. Sharma R. Gupta S. Majzoub A. Sabanegh E. (2017). Diagnostic Application of Oxidation-Reduction Potential Assay for Measurement of Oxidative Stress: Clinical Utility in Male Factor Infertility. Reprod. BioMedicine Online 34 (1), 48–57. 10.1016/j.rbmo.2016.10.008 27839743

[B7] Agarwal A. Rana M. Qiu E. AlBunni H. Bui A. D. Henkel R. (2018). Role of Oxidative Stress, Infection and Inflammation in Male Infertility. Andrologia 50 (11), e13126. 10.1111/and.13126 30569652

[B8] Agarwal A. Parekh N. Panner Selvam M. K. Henkel R. Shah R. Homa S. T. (2019). Male Oxidative Stress Infertility (MOSI): Proposed Terminology and Clinical Practice Guidelines for Management of Idiopathic Male Infertility. World J. Mens Health 37 (3), 296–312. 10.5534/wjmh.190055 31081299 PMC6704307

[B9] Aitken R. J. Baker M. A. (2006). Oxidative Stress, Sperm Survival and Fertility Control. Mol. Cell Endocrinol. 250 (1-2), 66–69. 10.1016/j.mce.2005.12.026 16412557

[B10] Aitken R. Smith T. Jobling M. Baker M. De Iuliis G. (2014). Oxidative Stress and Male Reproductive Health. Asian J. Androl. 16 (1), 31–38. 10.4103/1008-682X.122203 24369131 PMC3901879

[B11] Aitken R. J. (2017). Reactive Oxygen Species as Mediators of Sperm Capacitation and Pathological Damage. Mol. Reprod. Dev. 84 (10), 1039–1052. 10.1002/mrd.22871 28749007

[B12] Aitken R. J. (2020). Impact of Oxidative Stress on Male and Female Germ Cells: Implications for Fertility. Reproduction 159 (4), R189–R201. 10.1530/REP-19-0452 31846434

[B13] Alahmar A. T. Calogero A. E. Sengupta P. Dutta S. (2021). Coenzyme Q10 Improves Sperm Parameters, Oxidative Stress Markers and Sperm DNA Fragmentation in Infertile Patients with Idiopathic Oligoasthenozoospermia. World J. Mens Health 39 (2), 346–351. 10.5534/wjmh.190145 32009311 PMC7994657

[B14] Amaral A. Paiva C. Attardo Parrinello C. Estanyol J. M. Ballescà J. L. Ramalho-Santos J. (2014). Identification of Proteins Involved in Human Sperm Motility Using High-Throughput Differential Proteomics. J. Proteome Res. 13 (12), 5670–5684. 10.1021/pr500652y 25250979

[B15] Athayde K. S. Cocuzza M. Agarwal A. Krajcir N. Lucon A. M. Srougi M. (2007). Development of normal Reference Values for Seminal Reactive Oxygen Species and Their Correlation with Leukocytes and Semen Parameters in a fertile Population. J. Androl. 28 (4), 613–620. 10.2164/jandrol.106.001966 17409462

[B16] Baker M. A. Weinberg A. Hetherington L. Villaverde A.-I. Velkov T. Baell J. (2015). Defining the Mechanisms by Which the Reactive Oxygen Species By-Product, 4-Hydroxynonenal, Affects Human Sperm Cell Function1. Biol. Reprod. 92 (4), 108. 10.1095/biolreprod.114.126680 25673561

[B17] Barati E. Nikzad H. Karimian M. (2020). Oxidative Stress and Male Infertility: Current Knowledge of Pathophysiology and Role of Antioxidant Therapy in Disease Management. Cell. Mol. Life Sci. 77 (1), 93–113. 10.1007/s00018-019-03253-8 31377843 PMC11105059

[B18] Becatti M. Emmi G. Silvestri E. Bruschi G. Ciucciarelli L. Squatrito D. (2016). Neutrophil Activation Promotes Fibrinogen Oxidation and Thrombus Formation in Behçet Disease. Circulation 133 (3), 302–311. 10.1161/CIRCULATIONAHA.115.017738 26585672

[B19] Becatti M. Marcucci R. Gori A. M. Mannini L. Grifoni E. Alessandrello Liotta A. (2016). Erythrocyte Oxidative Stress Is Associated with Cell Deformability in Patients with Retinal Vein Occlusion. J. Thromb. Haemost. 14 (11), 2287–2297. 10.1111/jth.13482 27557753

[B20] Becatti M. Mannucci A. Barygina V. Mascherini G. Emmi G. Silvestri E. (2017). Redox Status Alterations during the Competitive Season in Élite Soccer Players: Focus on Peripheral Leukocyte-Derived ROS. Intern. Emerg. Med. 12 (6), 777–788. 10.1007/s11739-017-1653-5 28361355

[B21] Becatti M. Marcucci R. Mannucci A. Gori A. Giusti B. Sofi F. (2017). Erythrocyte Membrane Fluidity Alterations in Sudden Sensorineural Hearing Loss Patients: The Role of Oxidative Stress. Thromb. Haemost. 117 (12), 2334–2345. 10.1160/TH17-05-0356 29212121

[B22] Becatti M. Fucci R. Mannucci A. Barygina V. Mugnaini M. Criscuoli L. (2018). A Biochemical Approach to Detect Oxidative Stress in Infertile Women Undergoing Assisted Reproductive Technology Procedures. Int. J. Mol. Sci. 19 (2), 592. 10.3390/ijms19020592 PMC5855814 29462946

[B23] Benedetti S. Tagliamonte M. C. Catalani S. Primiterra M. Canestrari F. Stefani S. D. (2012). Differences in Blood and Semen Oxidative Status in fertile and Infertile Men, and Their Relationship with Sperm Quality. Reprod. BioMedicine Online 25 (3), 300–306. 10.1016/j.rbmo.2012.05.011 22818093

[B24] Birben E. Sahiner U. M. Sackesen C. Erzurum S. Kalayci O. (2012). Oxidative Stress and Antioxidant Defense. World Allergy Organ. J. 5 (1), 9–19. 10.1097/WOX.0b013e3182439613 23268465 PMC3488923

[B25] Bonanno O. Romeo G. Asero P. Pezzino F. M. Castiglione R. Burrello N. (2016). Sperm of Patients with Severe Asthenozoospermia Show Biochemical, Molecular and Genomic Alterations. Reproduction 152 (6), 695–704. 10.1530/REP-16-0342 27651518

[B26] Burton G. J. Jauniaux E. (2011). Oxidative Stress. Best Pract. Res. Clin. Obstet. Gynaecol. 25 (3), 287–299. 10.1016/j.bpobgyn.2010.10.016 21130690 PMC3101336

[B27] Cassina A. Silveira P. Cantu L. Montes J. M. Radi R. Sapiro R. (2015). Defective Human Sperm Cells Are Associated with Mitochondrial Dysfunction and Oxidant Production1. Biol. Reprod. 93 (5), 119. 10.1095/biolreprod.115.130989 26447142

[B28] Cayli S. Sakkas D. Vigue L. Demir R. Huszar G. (2004). Cellular Maturity and Apoptosis in Human Sperm: Creatine Kinase, Caspase-3 and Bcl-XL Levels in Mature and Diminished Maturity Sperm. Mol. Hum. Reprod. 10 (5), 365–372. 10.1093/molehr/gah050 15044602

[B29] Cito G. Becatti M. Natali A. Fucci R. Picone R. Cocci A. (2020). Redox Status Assessment in Infertile Patients with Non‐obstructive Azoospermia Undergoing Testicular Sperm Extraction: A Prospective Study. Andrologia 8 (2), 364–371. 10.1111/andr.12721 31654557

[B30] Dobrakowski M. Kasperczyk S. Horak S. Chyra-Jach D. Birkner E. Kasperczyk A. (2017). Oxidative Stress and Motility Impairment in the Semen of fertile Males. Andrologia 49 (10), e12783. 10.1111/and.12783 28261836

[B31] Dorostghoal M. Kazeminejad S. R. Shahbazian N. Pourmehdi M. Jabbari A. (2017). Oxidative Stress Status and Sperm DNA Fragmentation in fertile and Infertile Men. Andrologia 49 (10), e12762. 10.1111/and.12762 28124476

[B32] Du Plessis S. S. Agarwal A. Halabi J. Tvrda E. (2015). Contemporary Evidence on the Physiological Role of Reactive Oxygen Species in Human Sperm Function. J. Assist. Reprod. Genet. 32 (4), 509–520. 10.1007/s10815-014-0425-7 25646893 PMC4380893

[B33] Dutta S. Majzoub A. Agarwal A. (2019). Oxidative Stress and Sperm Function: A Systematic Review on Evaluation and Management. Arab J. Urol. 17 (2), 87–97. 10.1080/2090598X.2019.1599624 31285919 PMC6600059

[B34] Elkina Y. L. Atroshchenko M. M. Bragina E. E. Muronetz V. I. Schmalhausen E. V. (2011). Oxidation of Glyceraldehyde-3-Phosphate Dehydrogenase Decreases Sperm Motility. Biochem. Mosc. 76 (2), 268–272. 10.1134/s0006297911020143 21568861

[B35] Eruslanov E. Kusmartsev S. (2010). Identification of ROS Using Oxidized DCFDA and Flow-Cytometry. Methods Mol. Biol. 594, 57–72. 10.1007/978-1-60761-411-1_4 20072909

[B36] Gambera L. Stendardi A. Ghelardi C. Fineschi B. Aini R. (2019). Effects of Antioxidant Treatment on Seminal Parameters in Patients Undergoing *In Vitro* Fertilization. Arch. Ital. Urol. Androl. 91 (3), 187. 10.4081/aiua.2019.3.187 31577104

[B37] Gharagozloo P. Aitken R. J. (2011). The Role of Sperm Oxidative Stress in Male Infertility and the Significance of Oral Antioxidant Therapy. Hum. Reprod. 26 (7), 1628–1640. 10.1093/humrep/der132 21546386

[B38] Gholinezhad M. Aliarab A. Abbaszadeh-Goudarzi G. Yousefnia-Pasha Y. Samadaian N. Rasolpour-Roshan K. (2020). Nitric Oxide, 8-Hydroxydeoxyguanosine, and Total Antioxidant Capacity in Human Seminal Plasma of Infertile Men and Their Relationship with Sperm Parameters. Clin. Exp. Reprod. Med. 47 (1), 54–60. 10.5653/cerm.2020.00423 32079054 PMC7127900

[B39] Grotto D. Santa Maria L. D. Boeira S. Valentini J. Charão M. F. Moro A. M. (2007). Rapid Quantification of Malondialdehyde in Plasma by High Performance Liquid Chromatography-Visible Detection. J. Pharm. Biomed. Anal. 43 (2), 619–624. 10.1016/j.jpba.2006.07.030 16949242

[B40] Guo Y. Jiang W. Yu W. Niu X. Liu F. Zhou T. (2019). Proteomics Analysis of Asthenozoospermia and Identification of Glucose-6-Phosphate Isomerase as an Important Enzyme for Sperm Motility. J. Proteomics 208, 103478. 10.1016/j.jprot.2019.103478 31394311

[B41] Guz J. Gackowski D. Foksinski M. Rozalski R. Zarakowska E. Siomek A. (2013). Comparison of Oxidative Stress/DNA Damage in Semen and Blood of fertile and Infertile Men. PLoS One 8 (7), e68490. 10.1371/journal.pone.0068490 23874641 PMC3709910

[B42] Hallak J. Sharma R. K. Pasqualotto F. F. Ranganathan P. Thomas A. J. Jr Agarwal A. (2001). Creatine Kinase as an Indicator of Sperm Quality and Maturity in Men with Oligospermia. Urology 58 (3), 446–451. 10.1016/s0090-4295(01)01224-9 11549497

[B43] Halliwell B. (2007). Biochemistry of Oxidative Stress. Biochem. Soc. Trans. 35 (Pt 5), 1147–1150. 10.1042/BST0351147 17956298

[B44] Homa S. Vassiliou A. Stone J. Killeen A. Dawkins A. Xie J. (2019). A Comparison between Two Assays for Measuring Seminal Oxidative Stress and Their Relationship with Sperm DNA Fragmentation and Semen Parameters. Genes 10 (3), 236. 10.3390/genes10030236 PMC6471935 30893955

[B45] Hosseinzadeh Colagar A. Karimi F. Jorsaraei S. G. A. (2013). Correlation of Sperm Parameters with Semen Lipid Peroxidation and Total Antioxidants Levels in Astheno- and Oligoasheno- Teratospermic Men. Iran Red Crescent Med. J. 15 (9), 780–785. 10.5812/ircmj.6409 24616785 PMC3929810

[B46] Iommiello V. M. Albani E. Di Rosa A. Marras A. Menduni F. Morreale G. (2015). Ejaculate Oxidative Stress Is Related with Sperm DNA Fragmentation and Round Cells. Int. J. Endocrinol. 2015, 321901. 10.1155/2015/321901 25802519 PMC4352927

[B47] Kalezic A. Macanovic B. Garalejic E. Korac A. Otasevic V. Korac B. (2018). Level of NO/Nitrite and 3-Nitrotyrosine in Seminal Plasma of Infertile Men: Correlation with Sperm Number, Motility and Morphology. Chemico-Biological Interactions 291, 264–270. 10.1016/j.cbi.2018.07.002 29983398

[B48] Khosrowbeygi A. Zarghami N. (2007). Levels of Oxidative Stress Biomarkers in Seminal Plasma and Their Relationship with Seminal Parameters. BMC Clin. Pathol. 7, 6. 10.1186/1472-6890-7-6 17540046 PMC1906821

[B49] Koppers A. J. De Iuliis G. N. Finnie J. M. McLaughlin E. A. Aitken R. J. (2008). Significance of Mitochondrial Reactive Oxygen Species in the Generation of Oxidative Stress in Spermatozoa. J. Clin. Endocrinol. Metab. 93 (8), 3199–3207. 10.1210/jc.2007-2616 18492763

[B50] Kothari S. Thompson A. Agarwal A. du Plessis S. S. (2010). Free Radicals: Their Beneficial and Detrimental Effects on Sperm Function. Indian J. Exp. Biol. 48 (5), 425–435. 20795359

[B51] Kruk J. Aboul-Enein H. Y. Kładna A. Bowser J. E. (2019). Oxidative Stress in Biological Systems and its Relation with Pathophysiological Functions: The Effect of Physical Activity on Cellular Redox Homeostasis. Free Radic. Res. 53 (5), 497–521. 10.1080/10715762.2019.1612059 31039624

[B52] Lushchak V. I. (2014). Free Radicals, Reactive Oxygen Species, Oxidative Stress and its Classification. Chemico-Biological Interactions 224, 164–175. 10.1016/j.cbi.2014.10.016 25452175

[B53] Majzoub A. Agarwal A. (2018). Systematic Review of Antioxidant Types and Doses in Male Infertility: Benefits on Semen Parameters, Advanced Sperm Function, Assisted Reproduction and Live-Birth Rate. Arab J. Urol. 16 (1), 113–124. 10.1016/j.aju.2017.11.013 29713542 PMC5922223

[B54] Majzoub A. Arafa M. Mahdi M. Agarwal A. Al Said S. Al-Emadi I. (2018). Oxidation-reduction Potential and Sperm DNA Fragmentation, and Their Associations with Sperm Morphological Anomalies Amongst fertile and Infertile Men. Arab J. Urol. 16 (1), 87–95. 10.1016/j.aju.2017.11.014 29713539 PMC5922185

[B55] Makker K. Agarwal A. Sharma R. (2009). Oxidative Stress & Male Infertility. Indian J. Med. Res. 129 (4), 357–367. 19535829

[B56] Martins A. D. Agarwal A. (2019). Oxidation Reduction Potential: A New Biomarker of Male Infertility. Panminerva Med. 61 (2), 108–117. 10.23736/S0031-0808.18.03529-2 30990283

[B57] Morielli T. O'Flaherty C. (2015). Oxidative Stress Impairs Function and Increases Redox Protein Modifications in Human Spermatozoa. Reproduction 149 (1), 113–123. 10.1530/REP-14-0240 25385721 PMC5489333

[B58] Morrow J. D. (2005). Quantification of Isoprostanes as Indices of Oxidant Stress and the Risk of Atherosclerosis in Humans. Arterioscler Thromb. Vasc. Biol. 25 (2), 279–286. 10.1161/01.ATV.0000152605.64964.c0 15591226

[B59] Moscatelli N. Lunetti P. Braccia C. Armirotti A. Pisanello F. De Vittorio M. (2019). Comparative Proteomic Analysis of Proteins Involved in Bioenergetics Pathways Associated with Human Sperm Motility. Int. J. Mol. Sci. 20 (12), 3000. 10.3390/ijms20123000 PMC6627292 31248186

[B60] Muratori M. Tamburrino L. Marchiani S. Cambi M. Olivito B. Azzari C. (2015). Investigation on the Origin of Sperm DNA Fragmentation: Role of Apoptosis, Immaturity and Oxidative Stress. Mol. Med. 21 (1), 109–122. 10.2119/molmed.2014.00158 25786204 PMC4461587

[B61] Nallella K. P. Sharma R. K. Aziz N. Agarwal A. (2006). Significance of Sperm Characteristics in the Evaluation of Male Infertility. Fertil. Sterility 85 (3), 629–634. 10.1016/j.fertnstert.2005.08.024 16500330

[B62] Niki E. (2014). Biomarkers of Lipid Peroxidation in Clinical Material. Biochim. Biophys. Acta (Bba) - Gen. Subjects 1840 (2), 809–817. 10.1016/j.bbagen.2013.03.020 23541987

[B63] Nowicka-Bauer K. Nixon B. (2020). Molecular Changes Induced by Oxidative Stress that Impair Human Sperm Motility. Antioxidants 9 (2), 134. 10.3390/antiox9020134 PMC7070831 32033035

[B64] Nowicka-Bauer K. Lepczynski A. Ozgo M. Kamieniczna M. Fraczek M. Stanski L. (2018). Sperm Mitochondrial Dysfunction and Oxidative Stress as Possible Reasons for Isolated Asthenozoospermia. J. Physiol. Pharmacol. 69 (3), 403–417. 10.26402/jpp.2018.3.05 30149371

[B65] Ollero M. Gil-Guzman E. Lopez M. C. Sharma R. K. Agarwal A. Larson K. (2001). Characterization of Subsets of Human Spermatozoa at Different Stages of Maturation: Implications in the Diagnosis and Treatment of Male Infertility. Hum. Reprod. 16 (9), 1912–1921. 10.1093/humrep/16.9.1912 11527898

[B66] Pham-Huy L. A. He H. Pham-Huy C. (2008). Free Radicals, Antioxidants in Disease and Health. Int. J. Biomed. Sci. 4 (2), 89–96. 23675073 PMC3614697

[B67] Phaniendra A. Jestadi D. B. Periyasamy L. (2015). Free Radicals: Properties, Sources, Targets, and Their Implication in Various Diseases. Ind. J. Clin. Biochem. 30 (1), 11–26. 10.1007/s12291-014-0446-0 PMC4310837 25646037

[B68] Roychoudhury S. Sharma R. Sikka S. Agarwal A. (2016). Diagnostic Application of Total Antioxidant Capacity in Seminal Plasma to Assess Oxidative Stress in Male Factor Infertility. J. Assist. Reprod. Genet. 33 (5), 627–635. 10.1007/s10815-016-0677-5 26941096 PMC4870440

[B69] Sabeti P. Pourmasumi S. Rahiminia T. Akyash F. Talebi A. R. (2016). Etiologies of Sperm Oxidative Stress. Int. J. Reprod. Biomed. 14 (4), 231–240. 10.29252/ijrm.14.4.231 27351024 PMC4918773

[B70] Saleh R. A. Agarwal A. (2002). Oxidative Stress and Male Infertility: from Research Bench to Clinical Practice. J. Androl. 23 (6), 737–752. 10.1002/j.1939-4640.2002.tb02324.x 12399514

[B71] Saleh R. A. Agarwal A. Kandirali E. Sharma R. K. Thomas A. J. Nada E. A. (2002). Leukocytospermia Is Associated with Increased Reactive Oxygen Species Production by Human Spermatozoa. Fertil. Sterility 78 (6), 1215–1224. 10.1016/s0015-0282(02)04237-1 12477515

[B72] Salvolini E. Buldreghini E. Lucarini G. Vignini A. Di Primio R. Balercia G. (2012). Nitric Oxide Synthase and Tyrosine Nitration in Idiopathic Asthenozoospermia: an Immunohistochemical Study. Fertil. Sterility 97 (3), 554–560. 10.1016/j.fertnstert.2011.12.022 22244784

[B73] Shamsi M. B. Venkatesh S. Kumar R. Gupta N. P. Malhotra N. Singh N. (2010). Antioxidant Levels in Blood and Seminal Plasma and Their Impact on Sperm Parameters in Infertile Men. Indian J. Biochem. Biophys. 47 (1), 38–43. 21086753

[B74] Sharlip I. D. Jarow J. P. Belker A. M. Lipshultz L. I. Sigman M. Thomas A. J. (2002). Best Practice Policies for Male Infertility. Fertil. Sterility 77 (5), 873–882. 10.1016/s0015-0282(02)03105-9 12009338

[B75] Sharma R. K. Pasqualotto F. F. Nelson D. R. Thomas A. J. Jr Agarwal A. (1999). The Reactive Oxygen Species-Total Antioxidant Capacity Score Is a New Measure of Oxidative Stress to Predict Male Infertility*. Hum. Reprod. 14 (11), 2801–2807. 10.1093/humrep/14.11.2801 10548626

[B76] Sharma R. K. Pasqualotto A. E. Nelson D. R. Thomas A. J. Jr Agarwal A. (2001). Relationship between Seminal white Blood Cell Counts and Oxidative Stress in Men Treated at an Infertility Clinic. J. Androl. 22 (4), 575–583. 10.1002/j.1939-4640.2001.tb02217.x 11451354

[B77] Steiner A. Z. Hansen K. R. Barnhart K. T. Cedars M. I. Legro R. S. Diamond M. P. (2020). The Effect of Antioxidants on Male Factor Infertility: the Males, Antioxidants, and Infertility (MOXI) Randomized Clinical Trial. Fertil. Sterility 113 (3), 552–560. e3. 10.1016/j.fertnstert.2019.11.008 PMC7219515 32111479

[B78] Taken K. Alp H. H. Eryilmaz R. Donmez M. I. Demir M. Gunes M. (2016). Oxidative DNA Damage to Sperm Cells and Peripheral Blood Leukocytes in Infertile Men. Med. Sci. Monit. 22, 4289–4296. 10.12659/MSM.898631 27837200 PMC5110225

[B79] Vatannejad A. Tavilani H. Sadeghi M. R. Amanpour S. Shapourizadeh S. Doosti M. (2017). Evaluation of ROS-TAC Score and DNA Damage in Fertile Normozoospermic and Infertile Asthenozoospermic Males. Urol. J. 14 (1), 2973–2978. 28116742

[B80] Venkatesh S. Shamsi M. B. Deka D. Saxena V. Kumar R. Dada R. (2011). Clinical Implications of Oxidative Stress & Sperm DNA Damage in Normozoospermic Infertile Men. Indian J. Med. Res. 134 (3), 396–398. 21985826 PMC3193724

[B81] Vignini A. Nanetti L. Buldreghini E. Moroni C. Ricciardo-Lamonica G. Mantero F. (2006). The Production of Peroxynitrite by Human Spermatozoa May Affect Sperm Motility through the Formation of Protein Nitrotyrosine. Fertil. Sterility 85 (4), 947–953. 10.1016/j.fertnstert.2005.09.027 16580379

[B82] Wadhwa L. Priyadarshini S. Fauzdar A. Wadhwa S. N. Arora S. (2020). Impact of Vitamin D Supplementation on Semen Quality in Vitamin D-Deficient Infertile Males with Oligoasthenozoospermia. J. Obstet. Gynecol. India 70 (1), 44–49. 10.1007/s13224-019-01251-1 32030005 PMC6982618

[B83] Wagner H. Cheng J. W. Ko E. Y. (2018). Role of Reactive Oxygen Species in Male Infertility: An Updated Review of Literature. Arab J. Urol. 16 (1), 35–43. 10.1016/j.aju.2017.11.001 29713534 PMC5922220

[B84] Walters J. De Iuliis G. Nixon B. Bromfield E. (2018). Oxidative Stress in the Male Germline: A Review of Novel Strategies to Reduce 4-Hydroxynonenal Production. Antioxidants 7 (10), 132. 10.3390/antiox7100132 PMC6209867 30282920

[B85] Whittington K. Harrison S. C. Williams K. M. Day J. L. McLaughlin E. A. Hull M. G. R. (1999). Reactive Oxygen Species (ROS) Production and the Outcome of Diagnostic Tests of Sperm Function. Int. J. Androl. 22 (4), 236–242. 10.1046/j.1365-2605.1999.00174.x 10442296

[B86] Wolff H. (1995). The Biologic Significance of white Blood Cells in Semen. Fertil. Steril 63 (6), 1143–1157. 10.1016/s0015-0282(16)57588-8 7750580

[B87] Yoshida Y. Umeno A. Akazawa Y. Shichiri M. Murotomi K. Horie M. (2015). Chemistry of Lipid Peroxidation Products and Their Use as Biomarkers in Early Detection of Diseases. J. Oleo Sci. 64 (4), 347–356. 10.5650/jos.ess14281 25766928

